# Identification of the Alternative Splicing of the UL49 Locus of Human Cytomegalovirus

**DOI:** 10.1155/2015/280276

**Published:** 2015-03-19

**Authors:** Guang Yang, Wei Li, Wenzhen Liao, Xin Zhang, Yi Zou, Jianfeng Dai, Yueqin Li, Chunxia Jing, Tianhong Zhou

**Affiliations:** ^1^Department of Biotechnology, College of Life Science and Technology, Jinan University, Guangzhou 510632, China; ^2^Department of Parasitology, Medical School, Jinan University, Guangzhou 510632, China

## Abstract

The UL49 ORF of human cytomegalovirus (HCMV) is essential for viral replication; conserved among all herpes viruses; however, the function is unclear. Once the UL49 ORF was precisely deleted from the start to stop codon, the mutant did not yield infectious progeny. In this study, we find out many alternatively processed ESTs in UL49 locus in HCMV-infected cells, in which there are two novel transcription termination sites in UL49 locus. Most of these ESTs are rare transcripts that contain directed repeat sequences in the intron splicing regions. There is a typical GU-AG intron splicing site in UL49Y transcripts. The 1847 bp UL49Y cDNA spans an ORF from 335 to 1618 and encodes a putative protein of 427 amino acids with a predicted molecular mass of 47.1 kDa. All the new EST sequences and UL49Y cDNA sequence have been deposited in the GenBank database (GenBank Accession nos. GW314860-GW314900 and GU376796). This study provides us with very important clues for revealing the importance of the UL49 locus alternative splicing.

The human cytomegalovirus (HCMV), a *β*-herpes virus, is the most common cause of congenital infection and an important pathogen in immunocompromised individuals [1]. As the largest virus in the herpes virus family, the HCMV genome comprises ≈230 kb of double-stranded linear DNA [2]. Evaluation of the genome led to estimates of the number of protein-coding ORFs ranging from a maximum of 252 potentially functional ORFs that are conserved in different clinical isolates to a minimum of 165 ORFs that are conserved between HCMV clinical isolates [3–5]. Another significant uncertainty to the number of ORFs is our incomplete understanding of HCMV splicing. A variety of spliced mRNAs have been successfully identified [6–9], but so far there has been no exhaustive experimental search for spliced HCMV mRNAs. It is not possible to predict splice donors and acceptors with certainty.

HCMV UL49 ORF with unclear function is conserved among all herpes viruses. Once the UL49 ORF was precisely deleted from the start to stop codon, the mutant did not yield infectious progeny even after repeated transfection and extensive incubation [10]. Loss of replication due to a deletion of the whole UL49 locus may be caused by deletion of essential overlapping ORFs because the UL49 ORF overlaps with UL50 ORF and UL48.5 ORF nearby. The UL48, UL48.5, and UL50 ORFs all encode essential proteins. Since our recent study suggested a novel alternative transcript originating from the UL49 locus of the HCMV genome, which is different from the UL49 ORF and UL48.5 ORF, we still do not know whether there are new transcripts or not in UL49 ORF. For this reason we thoroughly examined HCMV-infected cells for more alternatively processed ESTs in UL49 locus.

Towne HCMV BAC containing a cassette for GFP eukaryotic expression was a gift from Professor Fenyong Liu, University of California, Berkeley [11]. Human dermal fibroblasts neonatal (HDFn) (Cascade Biologics) cells were electroporated with the Towne HCMV BAC DNA and an expression plasmid of the HCMV pp71 tegument protein, which can increase the infectivity of HCMV BAC DNA. Then we plated cells onto 75 cm^2^ flasks and observed for GFP expression under fluorescence microscopy. Infected cell culture media were cleared of cell debris by low-speed centrifugation and collected as stocks of cell-free virus.

HDFn cells were infected with the cell-free virus in Dulbecco's modified Eagle medium supplemented with 10% fetal calf serum (GIBCO/BRL). The final mixture will contain 10 *μ*g/mL gentamicin and 0.25 *μ*g/mL amphotericin B. Cells were cultured at 37°C in a humidified incubator with 5% CO_2_. RNA was extracted from infected HDFn cells cultured in six-well plates at various time points (2 h, 6 h, 12 h, 48 h, 72 h, and 96 h) after infection. To select for IE transcripts, cells were treated with the protein synthesis inhibitor cyclohexamide (100 *μ*g/mL) for 1 h prior to infection and throughout the 24 h infection period, when cells were harvested for RNA isolation. To select for E (early) transcripts, viral DNA replication inhibitor phosphonoformic acid (100 *μ*M) was added to the medium after the 24 h infection period, and cells were harvested 72 h after infection [11]. Total RNA of all samples was isolated using TRIzol (Invitrogen).

To define the 3′ end of the UL49 locus alternative mRNAs, we carried out the RACE method with 3′-Full RACE Core Sets (Takara) and all the primers have been listed in Table 1 and Figure 1. The cDNA template was synthesized with the Oligo dT-3sites Adaptor Primer of the 3′-Full RACE Core Set according to the manufacturer's instructions. The 1st PCR was performed with the 3′ RACE Outer primer (5′-TAC CGT CGT TCC ACT AGT GAT TT-3′) and Primer 1 in 50 *μ*L of the following reaction: 1 × LA PCR Buffer, 0.4 mM dNTP mixture, 0.2 *μ*M each primer, 2.5 U LA Taq polymerase, and 1 *μ*L of cDNA template. PCR amplification was done at 95°C for 5 min and followed by 30 cycles at 94°C for 30 s, 55°C for 1 min, and 72°C for 3 min. The 2nd PCR reactions and conditions were the same as the 1st PCR except for using the 3′RACE Inner primer (5′-CGC GGA TCC TCC ACT AGT GAT TTC ACT ATA GG-3′) and Primer 2, 1 *μ*L the outer PCR product as the template. Each sample was analyzed on a 1.5% agarose gel. Gel-purify of the PCR products were performed by using Gel Extraction Kit (E.Z.N.A.). Then all the isolated fragments were directly cloned into the TA-vector pMD18-T (TaKaRa) and sequenced using the sequencing Primer RV-M and Primer M13-47. Through the 3′-RACE PCR approaches, 10 novel cDNA fragments were cloned, with length from 188 bp to 1594 bp. These new EST sequences have been deposited in the GenBank database (GenBank Accession no. GW314860-GW314869) (Table 2). The various sequences comprised two groups. The group was characterized by the usage of a distinct alternative poly(A) sites within the 3′ untranslated region (3′UTR). Group A has two polyadenylation signals (AATAAA) located at 68947-68942 and 68648-68643 with the polyadenylation site at 68629. Group B has one polyadenylation signal (AATAAA) located at 69643-69638 with the polyadenylation site at 69610 (Figure 1).

According to the 3′ end of group A/B and the UL49 ORF 5′ end, primers were designed to do the nested PCR. The first-strand cDNA was synthesized using Takara 1st Strand cDNA Synthesis Kit. Total RNA samples (3 *μ*g) were reverse transcribed in a 10 *μ*L volume in the presence of 5 *μ*M Oligo dT Primer, 1 mM dNTP mixture. The reaction tube was incubated at 65°C for 5 min, followed by keeping in the ice. Then the following reagents were added to each reaction tube: 1 × PrimeScript Buffer, RNase Inhibitor 20 U, PrimeScript RTase 200 U, and RNase free DEPC H_2_O 4.5 *μ*L (Takara, Japan). Samples were incubated at 42°C for 60 min and 70°C for 15 min and then stored at 4°C. Then nested PCR was performed. For amplifying the transcripts from the UL49 ORF 5′ end to the group A, the outer PCR reactions volume is 50 *μ*L, which contains 1 *μ*L synthesized cDNA template, 1 × LA PCR Buffer, 0.4 mM dNTP mixture, 0.2 *μ*M Primer 3 and Primer 4, and 2.5 U LA Taq polymerase. Amplification conditions were 94°C for 30 s, 55°C for 1 min, and 72°C for 3.5 min for 30 cycles. The inner PCR reactions and conditions were the same as the outer PCR except for using Primer 5 and Primer 6, 1 *μ*L the outer PCR product as the template. To amplify the transcripts from the UL49 ORF 5′ end to group B, the outer PCR was performed with the Primer 3 and Primer 7, and inner PCR was performed with Primer 5 and Primer 8. The products of nested PCR amplification were inserted into pMD18-T cloning vector. The recombinant plasmid was transformed into* E. coli* DH5*α*. The amplicons were sequenced using Primer RV-M and Primer M13-47. A complete list of the cDNA clones and their positions relative to genomic DNA is listed in Tables 3 and 4. These new EST sequences have been deposited in the GenBank database (GenBank Accession no. GW314870-GW314900). We identified the situation of these transcripts in HCMV genome by BLAST software (http://blast.ncbi.nlm.nih.gov/Blast.cgi).

All these transcripts in UL49 locus have not been reported before. All the cDNA clones were acquired by using RACE and nested PCR. The results show the defects of bioinformatics methods in predicting alternatively spliced transcripts on one hand. In addition, nested PCR method was sensitive enough to find more transcripts. In fact, there were a lot of rare transcripts in this locus. The alternatively spliced UL49 variants detected suggest the complexity of transcription in the UL49 locus. We summarized all the novel transcripts, which was mapped with the R package software (ver.3.1.1) (Figure 4).

Most of these novel transcripts have directed repeat sequences in intron splicing regions, rather than typical RNA splice site GU-AG. The directed repeat sequences existed in many viruses, with different lengths and functions [12–16]. Further research was required in order to clarify the importance of directed repeat sequences in UL49 locus. All these transcripts in HCMV UL49 locus had not been found in past. It might be because of low abundances, which is similar to the transcripts in HCMV UL37 locus. Alternatively UL37 spliced variants are exceedingly low abundances relative to the UL37x1 unspliced transcript, some ~100 fold less and below detection by RT-PCR and gel detection, so the alternatively UL37 spliced variants cannot be detected by either S1 or Northern blot analysis [17, 18]. Consistent with these results, some UL49 spliced variant cDNAs were too low abundances to be rarely obtained in the HCMV-infected cells (Figure 2).

The functions of all the rare UL49 transcripts were unclear. We speculated that the functions of these transcripts might be for encoding virus proteins, or that they might play roles in regulating host cells or viral genes in the form of RNA. We will further do some research on the functions of these RNAs. Nonetheless, alternative processing of known HCMV transcripts results in the production of functionally different gene products. In the best-studied locus, differential processing of the major IE pre-mRNAs leads to the production of multiple spliced and polyadenylated RNAs. Moreover, Alwine has recently identified novel IE1 RNA splice variants, whose abundances differ during HCMV infection; however, their temporal expression is similar to that of IE1 mRNA. The products of the differentially spliced IE1 and IE2 transcripts differ in functions [17, 19].

Only the UL49X and UL49Y were detected from 2 h to 96 h after HCMV-infected cell and the other transcripts founded expressed temporally (Figure 2). UL49X and UL49Y can always be acquired, which might result from the fact that the UL49X and UL49Y have higher abundance and other transcripts have lower abundance. Although accurate splicing of the UL49X and UL49Y spliced junctions has been verified, we just obtained the EST fragments of UL49X and UL49Y, and the full-length UL49X and UL49Y cDNAs have not been cloned yet. Our next work is to obtain the UL49X and UL49Y full length cDNA.

The open reading frame was predicted by ORF finder program (http://www.ncbi.nlm.nih.gov/gorf/gorf.html). The full-length cDNA of UL49 began at 73134 bp and ended at 71047 bp, in which ORF began at 73043 bp and ended at 71331 bp. UL49X had the same 5′ end with UL49, but its 3′ end terminated at 68783 bp. A 2341 bp intron was deleted when UL49X cDNA transcript from the HCMV genome. So, it encoded a completely different protein from the UL49. The UL49X cDNA sequence has been deposited in the GenBank database (GenBank Accession no. GW314876). Because we could not search a complete ORF in UL49X by bioinformatics methods, we only analyzed the protein sequences of UL49Y with the complete ORF. The UL49Y cDNA sequence has been deposited in the GenBank database (GenBank Accession no. GU376796).

The 1847 bp UL49Y cDNA spanned an ORF from 335 to 1618 and encoded a putative protein of 427 amino acids with a predicted molecular mass of 47.1 kDa and an isoelectric point (pI) 9.35. There is an inframe stop codon at −72 prior to the first initiation codon. UL49Y cDNA contained a 95 bp intron and the intron conformed to the GU-AG rule (GGCTGgtgtg…tacagCATGGA). UL49Y is the truncated form of UL49, which lacks 143 aa in the 5′ end of UL49 (Figure 3).

We identified that UL49Y could encode a complete ORF by bioinformatics methods. But we need further experiments to prove whether it encodes a protein or not. UL49Y and UL49 have a common 3′ end, and current studies suggest that UL49 protein is an E (early) viral protein. In our experiment, we found that UL49Y began to express two hours after viral infection, which indicates that it is an IE (immediate early) protein. While UL49Y protein can continue to express till 96 hours, the different expression phase suggested that UL49Y may have distinct biological significance which is different from UL49. It is important for us to determine the functions of UL49Y.

Previous data reported that virus could not replicate if UL49 ORF was deleted [10]. Through bioinformatics analysis, we found that the deletion of UL49 also destroyed the UL50 ORF and UL49A ORF (Figure 1). And it may destroy the 3′UTR of UL48. We also found that the ORF of UL49Y was completely missing. Although so far we have not received full-length form of UL49X, but it is sure that the ORF of UL49X has also suffered damage when the UL49 ORF is deleted. We will examine the functions of different transcripts of this locus and reveal the molecular mechanisms why this locus is critical for virus replication. At meantime, we hope to learn the transcription situation of UL49 locus in other low passage CMV strains such as Merlin, TB40, and patient-derived clinical isolates.

Overall, we found two novel transcription termination sites in UL49 locus. In these two transcription termination sites, we found a large number of new transcripts, most of which are rare transcripts and contain directed repeat sequences in intron splicing regions. UL49X gene can express stably and has directed repeat sequences in intron splicing regions. There are typical GU-AG intron splicing sites in UL49Y transcripts. UL49Y might encode a full-length ORF. The above studies provide us with important clues for revealing the importance of the UL49 locus alternative splicing. This surprising transcript complexity makes the UL49 locus be the most complex of any known HCMV transcript [20].

## Figures and Tables

**Figure 1:**
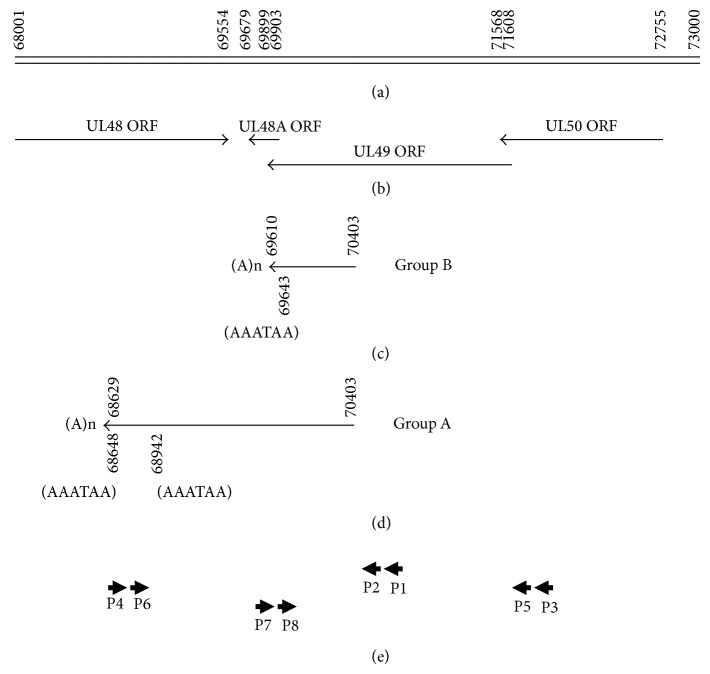
Organization and transcription summary of HCMV Towne UL49 locus. (a) Nucleotide positions correspond to the genome sequence under GenBank Accession no. AY315197.2. (b) ORF map summary of the UL48, UL48A, UL49, and UL50. (c) cDNAs and the location of polyadenylation signals and polyadenylation site are identified in group B. (d) cDNAs and the location of polyadenylation signals polyadenylation site are identified in group A. (e) Primers (Table 1) corresponding to the nucleotide position of HCMV Towne genome used in this paper.

**Figure 2:**
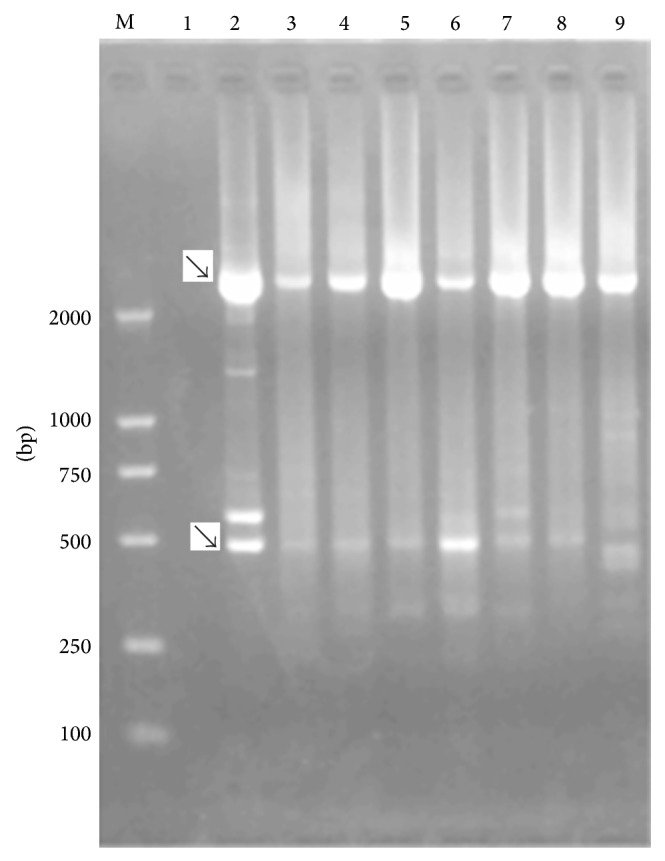
Agarose gel electrophoresis of differential transcripts of alternatively spliced UL49 cDNAs. Lane M: DNA Marker; Lane 1: cells uninfected by virus; Lanes 2 to 7: RT-PCR from HCMV-infected HDFn poly(A) RNA at 2, 6, 12, 48, 72, and 96 h postinfection, respectively; Lane 8: poly(A) RNA harvested at 72 h postinfection in the presence of inhibitor of DNA replication phosphonoformic acid (100 *μ*M); and Lane 9: poly(A) RNA harvested at 24 h postinfection in the presence of inhibitor of protein synthesis cyclohexamide (100 *μ*g/mL) 1 h prior to infection. UL49X and UL49Y were indicated by arrows.

**Figure 3:**
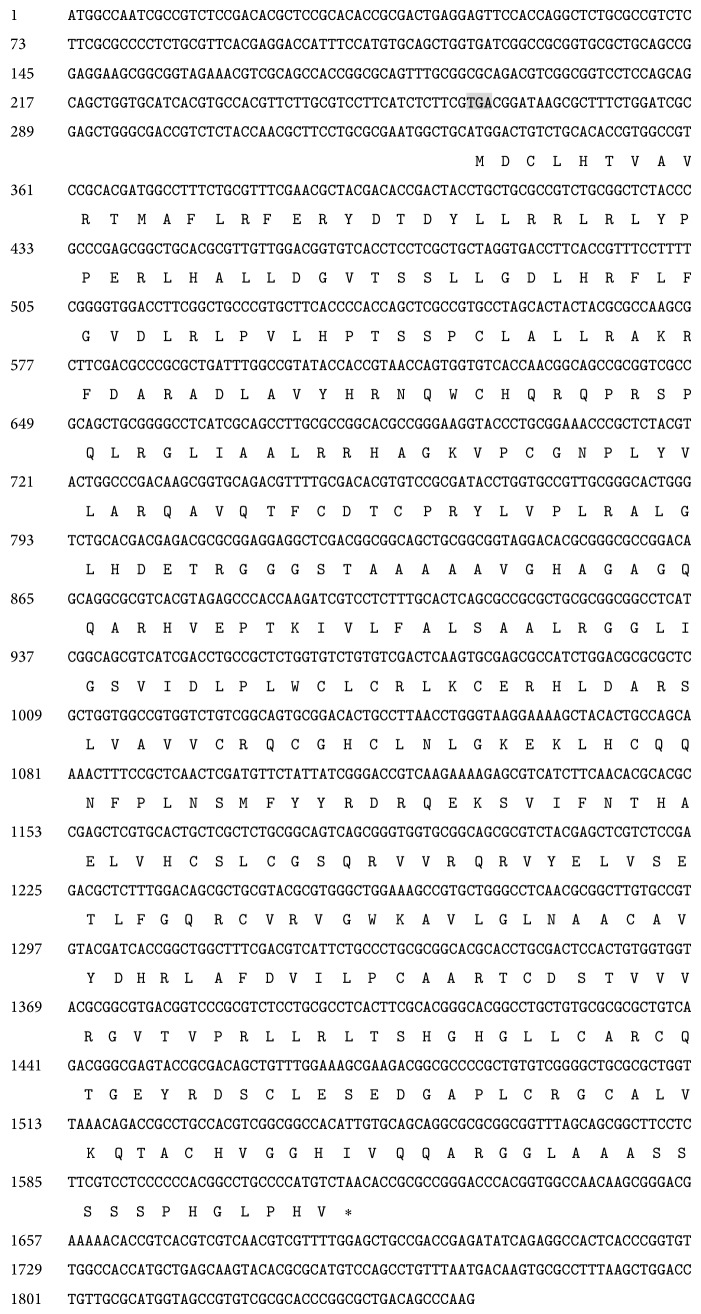
Nucleotide and deduced amino acid sequence of the UL49Y gene. Numbers on the left refer to the first nucleotide in each corresponding line. An upstream inframe stop codon (TGA) at 5′ to the start codon is shaded in gray. An asterisk indicates the stop codon. The DNA sequence has been deposited in the GenBank database (GenBank Accession no. GU376796).

**Figure 4:**
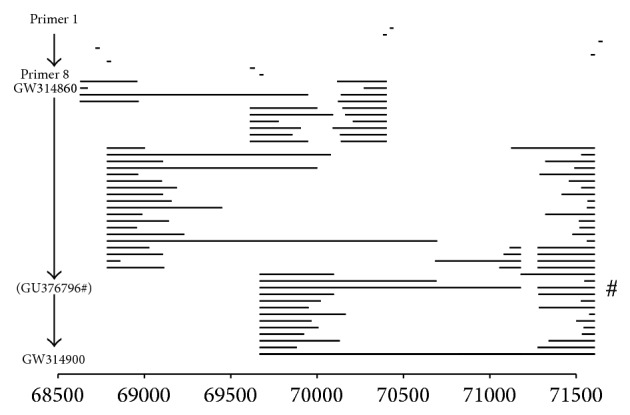
The summarize of all the alternative splicing transcripts founded in UL49 Locus. Primer 1–Primer 8 are the primers used in the research. The GW314850-GW314900 is the GenBank Accession number and the situations are the same in Tables 2, 3, and 4. The GU376796 and the pound symbol are the UL49Y. The numbers in *x*-axis mean the HCMV genome situation (AY315197.2).

**Table 1:** List of primers used in this paper.

Primer	Nucleotide sequence (5′-3′)	Corresponding to the nucleotide position of HCMV Towne genome
Primer 1	CTGCCAGCAAAACTTTCCGCT	70442–70422
Primer 2	TCGGGACCGTCAAGAAAAGAGCG	70403–70381
Primer 3	TGGTGCTGGCTCTCCTGCTGGTGC	71653–71630
Primer 4	GCATGTAGCCGACCTGCTGAAAGGC	68717–68741
Primer 5	ATGGCCAATCGCCGTCTCCGACAC	71608–71585
Primer 6	AGACCGCGACTTCCTCCGCATCCA	68783–68806
Primer 7	GTTACAAAACAACGTATCACTTTTACGG	69612–69639
Primer 8	CTTGGGCTGTCAGCGCCGGGTG	69667–69688
3′RACE Outer primer	TACCGTCGTTCCACTAGTGATTT	3′-Full RACE Core Sets kit supply
3′RACE Inner primer	CGCGGATCCTCCACTAGTGATTTCACTATAGG	3′-Full RACE Core Sets kit supply

**Table 2:** Sequences of novel HCMV UL49 alternatively spliced RNA fragments by 3′RACE.

Name of novel transcript	HCMV sequence (nt)^a^	Nested PCR product length (bp)	Intron length (bp)	Splice donor (**exon**/intron)^b^	Splice acceptor (intron/**extron**)^b^	GenBank accession number
UL49RA1	70403–70116; 68959–68629	619	1156	**GTCTCCTGCG**/CCTCACTTCG	GTCCTGTGCG/**TTGTAAATGA**	GW314860
UL49RA2	70403–70271; 68672–68629	177	1598	**TGGACAGCGC**/TGCGTACGCG	AGCCCAGCGC/**GTAGTCTAGG**	GW314861
UL49RA3	70403–70138; 69948–68626	1456	189	**GTACGCGGCG**/TGACGGTCCC	GCGCGCGGCG/**GTTTAGCAGC**	GW314862
UL49RA4	70403–70121; 68966–68626	624	1151	**GCGTCTCCTG**/CGCCTCACTT	TGTGGTCCTG/**TGCGTTGTAA**	GW314863
UL49RB1	70403–70146; 70001–69610	650	144	**CTGTGGTGGT**/ACGCGGCGTG	GCGCGCTGGT/**TAAACAGACC**	GW314864
UL49RB2	70403–70163; 70092–69610	724	70	**GCGGCACGCACCTGC**/GACTC	GGGCACGGCC/**TGCTGTGCGC**	GW314865
UL49RB3	70403–70207; 69777–69610	365	429	**TACGATCACC**/GGCTGGCTTT	GTTGGCCACC/**ATGCTGAGCA**	GW314866
UL49RB4	70403–70090; 69906–69610	611	183	**CGGGCACGGCCTGC**/TGTGC	CCACGGCCTGCC/**CCATGTCT**	GW314867
UL49RB5	70403–70133; 69857–69610	519	275	**CGGCGGGACG**/GTCCCGCGTC	AAGCGGGACG/**AAAAACACCG**	GW314868
UL49RB6	70403–70138; 69948–69610	605	189	**GTACGCGGCG**/TGACGGTCCC	GCGCGCGGCG/**GTTTAGCAGC**	GW314869

These RNA fragments were acquired by nested PCR with Primer 1 and 3′RACE Outer primer for outer PCR and Primer 2 and 3′RACE Inner prime for inner PCR. ^a^Numbering refers to HCMV genomic sequences (GenBank Accession no. AY315197.2). ^b^Underlines indicate direct repeat sequences.

**Table 3:** Novel sequences of HCMV UL49 alternatively spliced RNA fragments (group A).

Name of novel transcript	HCMV sequence (nt)^a^	Nested PCR product length (bp)	Intron length (bp)	Splice donor (**exon**/intron)^b^	Splice acceptor (intron/**extron**)^b^	GenBank accession number

UL49A1	71608–71124; 69003–68783	706	2120	**GTTTCGAACG**/CTACGACACC	TGTTCGAACG/**TGGAGGGCGG**	GW314870
UL49A2	71608–71530; 70080–68783	1377	1449	**CTCTTCGCGC**/CCCTCTGCGT	CTGTGCGCGC/**GCTGTCAGAC**	GW314871
UL49A3	71608–71321; 69108–68783	614	2212	**TCTGAATCGC**/GAGCTGGGCG	GGAGCGGCGC/**CAGACGCAGC**	GW314872
UL49A4	71608–71490; 70001–68783	1338	1488	**TGCAGCTGGT**/GATCGGCCGC	GCGCGCTGGT/**TAAACAGACC**	GW314873
UL49A5	71608–71288; 68963–68783	502	2324	**ACGCTTCCTG**/CGCGAATGGC	TGTGGTCCTG/**TGCGTTGTAA**	GW314874
UL49A6	71608–71457; 69101–68783	381	2355	**CGGAGGAAGC**/GGCGGTAGAA	CGCCAGACGC/**AGCGACGTTG**	GW314875
UL49X	71608–71530; 69188–68783	485	2341	**CTCTTCGCGC**/CCCTCTGCGT	GAACTCGCGC/**GATGGGTGGC**	GW314876
UL49A8	71608–71416; 69108–68783	519	2307	**TTTGCGGCGC**/AGACGTCGGC	GGAGCGGCGC/**CAGACGCAGC**	GW314877
UL49A9	71608–71565; 69157–68783	419	2407	**CCGCGACTGA**/GGAGTTCCAC	CAGAAACTGA/**ATATTGACTG**	GW314878
UL49A10	71608–71562; 69450–68783	715	2111	**CGACTGAGGA**/GTTCCACCAG	GCACTGAGGA/**CGTTCTGCGT**	GW314879
UL49A11	71608–71321; 68989–68783	495	2331	**TCTGAATCGC**/GAGCTGGGCG	GGGCGGTCGC/**AGCACGCGTA**	GW314880
UL49A12	71608–71515; 69142–68783	454	2372	**TGCGTTCACG**/AGGACCATTT	GACTGGCACG/**CGGTTGAAAG**	GW314881
UL49A13	71608–71519; 68957–68783	265	2561	**CCTCTGCGTT**/CACGAGGACC	CCTGTGCGTT/**GTAAATGACT**	GW314882
UL49A14	71608–71478; 69231–68783	580	2246	**TCGGCCGCGG** /TGCGCTGCAG	GCGGCCGCGG/**CCGCTCGATG**	GW314883
UL49A15	71608–71561; 70695–68783	1961	865	**GACTGAGGAG**/TTCCACCAGG	CGCGGAGGAG/**GCTCGACGGC**	GW314884
UL49A16	71608–71276; 71180–71114; 69028–68783	646	95^C^ 2085	**CGAATGGCTG**/GTGTGTCGGC **CTACGACACC**/GACTACCTGC	ACGTCTACAG/**CATGGACTGT** GTCCAACACC/**AGCCGACTGT**	GW314885
UL49A17	71608–71276; 71180–71079; 69107–68783	760	95^C^ 1971	**CGAATGGCTG**/GTGTGTCGGC **TCTACCCGCC**/CGAGCGGCTG	ACGTCTACAG/**CATGGACTGT** GAGCGGCGCC/**AGACGCAGCG**	GW314886
UL49A18	71608–71276; 71180–70683; 68860–68783	909	95^C^ 1822	**CGAATGGCTG**/GTGTGTCGGC **CGACGGCGGC**/AGCTGCGGCG	ACGTCTACAG/**CATGGACTGT** CTCCAGCGGC/**GTTTCGGTCC**	GW314887
UL49A19	71608–71276; 71180–71055; 69115–68783	792	95^C^ 1939	**CGAATGGCTG**/GTGTGTCGGC **CGTTGTTGGA**/CGGTGTCACC	ACGTCTACAG/**CATGGACTGT** CGGTGTTGGA/**GCGGCGCCAG**	GW314888

These RNA fragments were amplified by nested PCR with Primer 3 and Primer 4 for outer PCR and Primer 5 and Primer 6 for inner PCR. ^a^Numbering refers to HCMV genomic sequences (GenBank Accession no. AY315197.2). ^b^Underlines indicate direct repeat sequences. ^C^Introns conform to the GU-AG rule.

**Table 4:** Sequences of novel HCMV UL49 alternatively spliced RNA fragments (group B).

Name of novel transcript	HCMV sequence (nt)^a^	Nested PCR product length (bp)	Intron length (bp)	Splice donor (**exon**/intron)^b^	Splice acceptor (intron/**extron**)^b^	GenBank accession number
UL49B1	71608–71179; 70097–69667	861	1081	**GTCTACAGCA**/TGGACTGTCT	CGCACGGGCA/**CGGCCTGCTG**	GW314889
UL49B2	71608–71548; 70691–69667	1086	856	**CACCAGGCTC**/TGCGCCGTCT	GAGGAGGCTC/**GACGGCGGCA**	GW314890
UL49Y	71608–71276; 71180–69667	1847	95^C^	**CGAATGGCTG**/GTGTGTCGGC	ACGTCTACAG/**CATGGACTGT**	GU376796
UL49B4	71608–71282; 70097–69667	758	1184	**CCTGCGCGAA**/TGGCTGGTGT	CGCACGGGCA/**CGGCCTGCTG**	GW314891
UL49B5	71608–71527; 70021–69667	437	1505	**TTCGCGCCCC**/TCTGCGTTCA	GCGCCCCGCTG/**TGTCGGGGCT**	GW314892
UL49B6	71608–71284; 69951–69667	610	1332	**TTCCTGCGCG**/AATGGCTGGT	CAGGCGCGCG/ **GCGGTTTAGC**	GW314893
UL49B7	71608–71576; 70166–69667	533	1409	**CGCTCCGCAC**/ACCGCGACTG	CGGCACGCAC/**CTGCGACTCC**	GW314894
UL49B8	71608–71501; 69968–69667	410	1532	**CCATTTCCAT**/GTGCAGCTGG	GCGGCCACAT/**TGTGCAGCAG**	GW314895
UL49B9	71608–71543; 70007–69667	407	1535	**GGCTCTGCGC**/CGTCTCTTCG	GGGGCTGCGC/**GCTGGTTAAA**	GW314896
UL49B10	71608–71533; 69925–69667	335	1607	**CGTCTCTTCG**/CGCCCCTCTG	TTCCTCTTCG/**TCCTCCCCCC**	GW314897
UL49B11	71608–71341; 70131–69667	733	1209	**TTCGTGACGG**/ATAAGCGCTT	GGCGTGACGG/**TCCCGCGTCT**	GW314898
UL49B12	71608–71276; 71180–70822; 69882–69667	908	95^C^ 939	**CGAATGGCTG**/GTGTGTCGGC **CACGCCGGGA**/AGGTACCCTG	ACGTCTACAG/**CATGGACTGT** CGCGCCGGGA/**CCCACGGTGG**	GW314899
UL49B13	71608–69667	1942	—	—	—	GW314900

^a^Numbering refers to HCMV genomic sequences (GenBank Accession no. AY315197.2).

^b^Underlines indicate direct repeat sequences. ^C^Introns conform to the GT-AG rule.

—: There is no intron in the transcript of UL49B13.
